# Verrucous Carcinoma in a Giant Cutaneous Horn: A Case Report and Literature Review

**DOI:** 10.1155/2020/7134789

**Published:** 2020-01-31

**Authors:** Sudha Shahi, Tika Ram Bhandari, Tridip Pantha

**Affiliations:** ^1^Otorhinolaryngology Head and Neck Surgery, National Academy of Medical Sciences, Bir Hospital, Kathmandu, Nepal; ^2^General Surgery, People's Dental College and Hospital, Kathmandu, Nepal

## Abstract

**Background:**

A cutaneous horn is a common clinical entity which usually presents as a cutaneous lesion. Because of its subtle nature, patients usually tend to present late unless the lesion is big or complications develop. Because of its resemblance to animal horn, it has been given the term “horn.” Cutaneous horn seems to have a remarkable history. Though cutaneous horn is benign most of the times, chances of malignancy (20–25%) should be kept in mind. Old age, giant cutaneous horn carries more chances of transformation into malignancy like in our case. Thus, early diagnosis and treatment is required in all cases. *Case Presentation*. We report a case of a 74-year-old farmer with a cutaneous projection measuring ∼8 × 5 × 3 cm^3^ over the medial surface of the right pinna for 1 year. It started as a small projection which was progressively enlarging. The primary reason behind him presenting to us was cosmetic reason since it resembled an animal horn. The projection was not associated with pain or similar lesions anywhere else in body. Understanding the malignancy risks and the cosmetic benefits, he was planned for excision biopsy of the horn. He had no systemic signs of malignancy. Histopathological reports were consistent with malignancy.

**Conclusions:**

Cutaneous horns are usually benign lesions and mostly found in the head and neck region. Because of the chances of malignancy, cutaneous horns should undergo surgical removal and biopsy for early and definitive diagnosis and management.

## 1. Introduction

A cutaneous horn is a hard, conical, dense, hyperkeratotic cutaneous lesion. There were accounts of giant cutaneous horns in the sixteenth and seventeenth centuries which might have been due to lack of knowledge and lack of health facilities. Numerous natural and supernatural theories have been discussed in history regarding their etiology. The late eighteenth century marks the characterization of the disease as a medical disorder by surgeons Everard Home and his brother-in-law John Hunter in London [1, 2]. Though cases of small cutaneous horns with verrucous carcinoma have been reported in literature, a giant cutaneous horn is very rare and typical presentation. So, we present a very typical case with a giant horn in his right pinna resembling an animal horn, to be more specific.

## 2. Case Presentation

**Figure 1:**
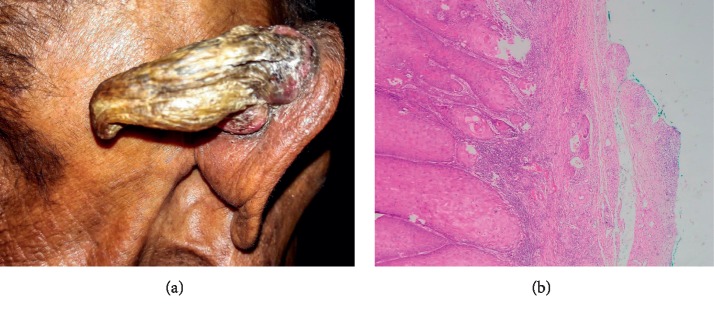
(a) Giant cutaneous horn over the medial aspect of right pinna. (b) Histopathological slide of the surgical specimen.

A 74-year-old farmer presented with a cutaneous projection over medial surface of his right pinna for 1 year. It started as a small cutaneous projection which was progressively enlarging. He denied any history of pain but had discomfort over the pinna. There was no history of discharge from the projection. There was no history of hearing loss, tinnitus. There was no history of similar lesions or any other mass in other parts of his body. He did not give any history of weight loss and loss of appetite. There was no history of significant medical illness, trauma to ear or surgery in the past. He was a nonsmoker and did not drink alcohol. He did not give history of similar lesions in any of his family members. On examination, his vitals were within normal limits. Systemic examination revealed no abnormality. On examination of the lesion, there was a woody hard, nontender cutaneous projection, measuring ∼8 × 5 × 3cm^3^ over the medial surface of right pinna (Figure 1(a)). Skin surrounding the projection was normal with no ulceration or discharge. There was no overlying pulsation. The projection was fixed to underlying structure. There was no enlargement of regional lymph nodes. He was posed the diagnosis of cutaneous horn. The next day, he underwent complete excision of the cutaneous horn with 5 mm margin with postauricular local advancement flap under local anesthesia. Postoperative period was uneventful. He was discharged with oral antibiotics and analgesics. Histopathological report was consistent with verrucous squamous cell carcinoma (Figure 1(b)) with negative margins. Thus, he was kept under regular follow-up for following 6 months. During the period, the operated site healed well. He had no other complaints related to the surgical procedure. He showed no signs of local recurrence.

## 3. Discussion

Cutaneous horn, also known as cornu cutaneum, is a form of hypertrophic senile keratosis in which the horny layer accumulates and adheres resulting to horn. In around 20% to 25% of cases malignant transformation into squamous cell carcinoma has been found [3]. The earliest documented case of cornu cutaneum was of Mrs. Margaret Gryffith, an elderly Welsh woman in London in 1588 [4]. The term cutaneous horn was coined because of its resemblance to an animal horn [5]. However, both are histologically different. Animal horns are composed of superficial hyperkeratotic epidermis, dermis, and a centrally positioned bone, while human cutaneous horns contain cystic structures lined by trichilemmal-type epithelium and lack centrally positioned bone. Several kinds of cutaneous horns have been described: (1) filiform horn, (2) papillomatous horn, and (3) verrucous horn. They differ from one another in appearance, causation, and histologic features [5, 6]. Base of a cutaneous horn may be associated with lesions mainly including squamous cell carcinoma, viral warts, actinic keratosis, keratoacanthoma, Bowen's disease, seborrheic keratosis, basal-cell carcinoma, and Kaposi sarcoma. Other less common are epidermal nevus, ichthyosis hystrix, verruca vulgaris, actinic keratosis, other precancerous keratosis, seborrheic keratosis, molluscum contagiosum, trichilemmal cysts, or epidermoid cysts [3, 7]. According to a study of Yu et al., cutaneous horns were more common in the sun-exposed areas of the body. The study also found that 61% of the cutaneous horns were derived from benign lesions and 39% from premalignant (23.2%) or malignant (15.7%) epidermal lesions. Clinically, it might be difficult to differentiate between benign and malignant lesions. However, malignant horns might be more commonly associated with male sex, older age, photo-exposed areas, and giant horns [8]. A study by Mantese et al. also showed squamous cell carcinoma in 94% of the malignant lesions. There was female preponderance in (64.86%) [9].

Early diagnosis and treatment is mandatory to prevent the risk of transformation to malignancy and the psychosocial stress owing to the bizarre presentation. Surgical excision remains the treatment of choice in most cases [10]. Surgical excision with 3 mm to 1 cm margins is required in most cases since there is association with malignancy [11]. Histopathological review is always necessary to rule out malignancy. Other possible options for treatment include electro cautery, cryotherapy, carbon dioxide, and Nd YAG laser. Cryosurgery may be used as an alternative treatment, although it is not recommended because it does not preserve the specimen for histopathology. These alternative methods of treatment are mostly preferred in cases where the lesion is small and there is low grade of suspicion of malignancy [12]. Sunscreens might have a preventive role in case of lesions commonly seen in the sun-exposed areas [9]. In our case, there was high suspicion of malignancy. Thus, complete surgical excision with clear margins was adopted as the treatment of choice. He was then kept under regular follow-up. However, the patient was lost to follow-up after 6 months.

## 4. Conclusion

Giant cutaneous horns might present as small asymptomatic lesions. Despite the fact that majority of lesions are benign, the risk of malignant transformation should not be ignored. The standard treatment should be excision biopsy with adequate margins. We have reported our case owing to its typical presentation, a close resemblance of an animal horn and its association with malignancy. Very few cases of “giant cutaneous horn” have been found in literature. We have tried to keep our focus over the unavoidable risk of malignancy which should be addressed on time to avoid physical and psychosocial stress to the patient and the attending family members.

## Abbreviations

Nd: Neodymium-doped
YAG: Yttrium aluminium garnet.

## References

[1] Home E. (1791). VI. observations on certain horny excrescences of the human body. *Philosophical Transactions of the Royal Society of London*.

[2] Bondeson J. (2001). Everard Home, John hunter, and cutaneous horns. *The American Journal of Dermatopathology*.

[3] Sandbank M. (1971). Basal cell carcinoma at the base of cutaneous horn (cornu cutaneum). *Archives of Dermatology*.

[4] Castillo D., Zerpa O., Loyo N., López C., Oliver M. (2002). Histopatología del cuerno cutaneo: estudio retrospectivo de 77 casos. *Dermatol Venez*.

[5] Montgomery D. W. (1941). Cornu cutaneum. *Archives of Dermatology*.

[6] Michal M., Bisceglia M., Di Mattia A. (2002). Gigantic cutaneous horns of the scalp. *The American Journal of Surgical Pathology*.

[7] Vano-Galvan S., Sanchez-Olaso A. (2008). Squamous-cell carcinoma manifesting as a cutaneous horn. *New England Journal of Medicine*.

[8] Yu R. C. H., Pryce D. W., Macfarlane A. W., Stewart T. W. (1990). A histopathological study of 643 cutaneous horns. *British Journal of Dermatology*.

[9] Mantese S. A. d. O., Diogo P. M., Rocha A., Berbert A. L. C. V., Ferreira A. K. M., Ferreira T. C. (2010). Corno cutâneo: estudo histopatológico retrospectivo de 222 casos. *Anais Brasileiros de Dermatologia*.

[10] Leppard W., Loungani R., Saylors B., Delaney K. (2014). Mythology to reality: case report on a giant cutaneous horn of the scalp in an African American female. *Journal of Plastic, Reconstructive & Aesthetic Surgery*.

[11] Wollina U., Schönlebe J. (2010). Giant ker toacanthoma-like cutaneous horn of the upper leg: a case report. *Acta Dermatoven, Alpina Pannonica et Adriatica*.

[12] Lowe F. C., McCullough A. R. (1985). Cutaneous horns of the penis: an approach to management. *Journal of the American Academy of Dermatology*.

